# Therapeutic Alliance With a Fully Automated Mobile Phone and Web-Based Intervention: Secondary Analysis of a Randomized Controlled Trial

**DOI:** 10.2196/mental.4656

**Published:** 2016-02-25

**Authors:** Janine Clarke, Judith Proudfoot, Alexis Whitton, Mary-Rose Birch, Megan Boyd, Gordon Parker, Vijaya Manicavasagar, Dusan Hadzi-Pavlovic, Andrea Fogarty

**Affiliations:** ^1^ School of Psychiatry UNSW Australia Sydney Australia; ^2^ Black Dog Institute UNSW Australia Sydney Australia

**Keywords:** therapeutic alliance, e-therapy, Internet interventions, depression, computerized cognitive behavior therapy

## Abstract

**Background:**

Studies of Internet-delivered psychotherapies suggest that clients report development of a therapeutic alliance in the Internet environment. Because a majority of the interventions studied to date have been therapist-assisted to some degree, it remains unclear whether a therapeutic alliance can develop within the context of an Internet-delivered self-guided intervention with no therapist support, and whether this has consequences for program outcomes.

**Objective:**

This study reports findings of a secondary analysis of data from 90 participants with mild-to-moderate depression, anxiety, and/or stress who used a fully automated mobile phone and Web-based cognitive behavior therapy (CBT) intervention called “myCompass” in a recent randomized controlled trial (RCT).

**Methods:**

Symptoms, functioning, and positive well-being were assessed at baseline and post-intervention using the Depression, Anxiety and Stress Scale (DASS), the Work and Social Adjustment Scale (WSAS), and the Mental Health Continuum-Short Form (MHC-SF). Therapeutic alliance was measured at post-intervention using the Agnew Relationship Measure (ARM), and this was supplemented with qualitative data obtained from 16 participant interviews. Extent of participant engagement with the program was also assessed.

**Results:**

Mean ratings on the ARM subscales were above the neutral midpoints, and the interviewees provided rich detail of a meaningful and collaborative therapeutic relationship with the myCompass program. Whereas scores on the ARM subscales did not correlate with treatment outcomes, participants’ ratings of the quality of their emotional connection with the program correlated significantly and positively with program logins, frequency of self-monitoring, and number of treatment modules completed (*r* values between .32-.38, *P*≤.002). The alliance (ARM) subscales measuring perceived empowerment (*r*=.26, *P*=.02) and perceived freedom to self-disclose (*r*=.25, *P*=.04) also correlated significantly in a positive direction with self-monitoring frequency.

**Conclusions:**

Quantitative and qualitative findings from this analysis showed that a positive therapeutic alliance can develop in the Internet environment in the absence of therapist support, and that components of the alliance may have implications for program usage. Further investigation of alliance features in the Internet environment and the consequences of these for treatment outcomes and user engagement is warranted.

**Trial Registration:**

Australian New Zealand Clinical Trials Registry Number (ACTRN): 12610000625077; https://www.anzctr.org.au/Trial/Registration/TrialReview.aspx?id=335772&isReview=true (Archived by WebCite at http://www.webcitation.org/6efAc5xj4).

##  Introduction

Internet delivered psychotherapy for common mental health problems can assist with reducing the problem of unmet treatment need by overcoming barriers to access (including financial, temporal, and geographic constraints), and offering advantages of user-privacy and 24-hour availability [1]. In the case of depression and anxiety, interventions delivered via the Internet are popular with users [2], cost efficient, and clinically effective, with outcomes equivalent to face-to-face psychological therapies [3-5].

Much of the variance in outcomes of face-to-face psychotherapy has been attributed to the therapeutic alliance, defined as a non-specific treatment factor reflecting the extent of collaboration, purposeful action, and emotional connection between a client and therapist [6]. Considered the “quintessential integrative variable” of therapy [7] that enables patients to “accept, follow, and believe in treatment” [8], client-derived ratings of the therapeutic alliance have been associated with positive outcomes in face-to-face therapy irrespective of the type of psychological treatment, study design, and outcome measure used [8,9]. In some studies, therapeutic alliance accounts for approximately 50% of the effect size [10]. Positive associations have also been found between the therapeutic alliance and treatment engagement [11,12].

A tendency for humans to respond socially to computing technology in the same way as they respond to other humans has been well documented [13], and anthropomorphism (ie, the attribution of human qualities to inanimate objects) is widely recognized as integral to the successful design and use of information technologies [14,15], including in the therapeutic context [16,17]. Nevertheless, therapy provided in the Internet environment has been criticized for its limited capacity to facilitate a therapeutic relationship due to reduced responsiveness to nonverbal interpersonal cues, limited ability to provide reassurance and clarification of misunderstandings, potential for conflicts of interest, and difficulty providing timely corrective feedback [18-20]. By and large, however, the available evidence tends not to support these criticisms [21,22]. On the contrary, recent reviews show that client ratings of the therapeutic alliance in Internet-delivered interventions are generally equivalent to ratings in face-to-face therapy [21,22], suggesting that development of a therapeutic relationship during Internet-based psychotherapy is indeed possible. However, there is no consensus as to whether this has implications for treatment outcomes [21].

The majority of evidence relating to the therapeutic alliance in Internet-delivered interventions derives from studies of interventions that are therapist-assisted to some extent. The nature of the client-therapist interaction varies widely between existing programs, in terms of frequency (ie, number of interactions), nature (eg, provision of therapeutic support, technical support, and/or feedback on therapeutic tasks), modality (ie, email versus SMS text message (short message service)), and timeliness (ie, synchronous versus asynchronous) [22-25]. Nevertheless, all programs have some degree of overt therapist input into a client’s treatment. In some studies, program users have even been provided with the name, photo identification, and biographical details of the assisting therapist [23]. This being the case, it is difficult to conclude whether existing findings of a positive therapeutic alliance in Internet-delivered psychotherapy reflect the quality of clients’ working relationships with the therapists involved or with the Internet programs themselves. Furthermore, it remains unclear as to whether alliance features can develop in the Internet environment in the absence of therapist assistance.

To the best of our knowledge, only one study has examined whether a therapeutic alliance can develop in a completely automated and self-guided Internet-delivered intervention without therapist input. Ormrod et al [26] used the Agnew Relationship Measure (ARM) [27] to examine the therapeutic alliance in a pilot study (N=16) of *Beating the Blues*, a Web-based cognitive behavior therapy (CBT) intervention for depression and anxiety. The ARM assesses 5 dimensions of the client-therapist relationship: bond, partnership, confidence, openness, and client initiative. On average, participants’ perception of the strength of their alliance with the program was positive, although ratings of the alliance were lower than those noted for face-to-face cognitive behavioral therapy (CBT). These pilot data are tentative, however, and require replication in a larger and more rigorously designed study.

In a recently completed randomized controlled trial (RCT), we reported significant symptom and functional outcome gains for people with mild-to-moderate depression, anxiety, and stress who used a fully automated mobile phone and Web intervention with no therapist input, known as “myCompass” [28] (ACTRN 12610000625077). This paper reports outcomes of a secondary objective of the RCT; namely to explore the role of the therapeutic alliance in this type of intervention. Specifically, we explored whether (1) participants reported a therapeutic alliance with the intervention; (2) participants’ ratings of the therapeutic alliance were associated with symptom and functional gains and improved positive well-being; and (3) ratings of therapeutic alliance features were associated with participants’ level of program engagement. Whereas previous studies of the therapeutic alliance have used predominantly quantitative methods, we also utilized interview methodology to further examine in qualitative detail the nature and form of participants’ reported alliance with the mobile phone and Web-based intervention.

## Methods

### Participants and Procedure

The myCompass RCT examined the effectiveness of a fully automated and self-guided psychological treatment that is delivered via the Internet to mobile and stationary technology devices for improving mental health symptom and functional outcomes. Participants in the RCT were 720 community volunteers with self-reported mild-to-moderate depression, anxiety, and/or stress symptoms who were recruited nationally in Australia between October 2011 and March 2012 via Internet, radio, and print media advertising. Following baseline assessment, participants were randomly allocated to the myCompass intervention, an attention control program, or a waitlist control condition, for 7 weeks. Subsequent assessments were conducted online on completion of the intervention phase (8 weeks) and at 20 weeks. The design and recruitment procedures for the myCompass RCT have previously been reported in greater detail [28].

Quantitative data for this secondary analysis was derived from participants randomly allocated to receive the myCompass intervention. This was supplemented by qualitative data provided by a purposively selected sample that completed the post-intervention questionnaire and agreed, via email, to participate in a semi-structured telephone interview with one of the authors (MAB). A sampling to saturation recruitment method was used in which data collection continued until no new themes emerged from the interviews [29].

The RCT was approved by the Human Research Ethics Committee of the University of New South Wales, Sydney, Australia (HREC 10019) and registered with the Australian and New Zealand Clinical Trials Registry (ACTRN 12610000625077). The CONSORT-EHEALTH checklist is provided as Multimedia Appendix 1.

### The myCompass Mobile Phone and Web Intervention

myCompass is a fully automated public health CBT-based intervention for the treatment of mild-to-moderate depression, anxiety, and stress [30]. The program is completely self-guided with no therapist input, and is accessible from any Internet-enabled mobile phone, tablet, or computer.

The myCompass program assesses users’ self-reported symptoms on registration and then provides 24/7 access to a personalized intervention that includes real-time, self-monitoring of moods and behaviors (via mobile phone, tablet, or computer), and interactive, evidence-based psychotherapeutic modules (via tablet and computer). In addition, users can schedule SMS text message (short message service) or email reminders to facilitate self-monitoring, receive and print graphical feedback about their self-monitoring alongside contextual information on their phone or computer (to monitor change and assist identification of triggers), and elect to receive helpful facts, mental health care tips or motivational statements by text message or email. Registering to use the program is free, and users are not billed for the text messages they receive. A detailed description of the myCompass intervention is provided in Proudfoot et al [28].

### Data Collection

#### Quantitative Measures

Participants in the RCT completed standardized and validated measures of mental health symptoms, work and social functioning, and positive psychological well-being at baseline, post-intervention, and follow-up, and the therapeutic alliance at post-intervention.

The Depression, Anxiety and Stress Scales (DASS) is widely used to measure the extent to which a person experienced symptoms of depression, anxiety, and stress over the previous week [31]. Total scores on the DASS range from 0 to 126 and subscale scores range from 0 to 42, with higher scores indicating greater symptom severity.

The Work and Social Adjustment Scale (WSAS) assesses the impact of mental health problems on daily functioning in 5 domains: work, social leisure activities, private leisure activities, home-management, and personal relationships [32]. Scores on the WSAS range from 0 to 40, with higher scores indicating poorer adjustment.

The Mental Health Continuum-Short Form (MHC-SF) measures positive mental health defined as the presence of positive feelings (emotional well-being), and positive functioning in individual life (psychological well-being) and community life (social well-being) [33]. Total scores and subscale scores on the MHC-SF range from 0 to 5, with higher scores indicating higher levels of positive mental health.

The Agnew Relationship Measure (ARM) uses 28 items to measure 5 elements of the client-therapist relationship: the affective element (bond), extent of mutual collaboration and engagement (partnership), perceived professional competence (confidence), perceived freedom to disclose personal concerns (openness), and empowerment (client initiative) [27]. Subscale scores range from 1 to 7, with higher scores indicating a stronger and more positive client-therapist alliance. In line with Ormrod et al [26], and to facilitate comparison of our data with theirs, ARM items were modified in the present study by replacing the word “therapist” with “program.”

Participant engagement with the myCompass program was measured using the following 3 indices: (1) number of program interactions (ie, logins), (2) number of modules completed, and (3) frequency of self-monitoring [34].

#### Qualitative Measures

Participant interviews were semi-structured and asked about the non-specific or “common” treatment factors identified in previous studies as contributing to development, persistence, and quality of the therapeutic alliance in face-to-face psychotherapy (Table 1). The interview comprised 16 open-ended questions and commenced with the question “Can you tell me what you liked most about the myCompass program?” Interview questions were theory-based, derived from the ARM, and the Model of Common Factors (MCFs) [35,36] (Table 1). Consent for participation and tape recording was obtained before each interview.

**Table 1:** Components of the semi-structured interview and their theoretical bases.

Broad concept	Agnew Relationship Measure (ARM) [27]	Model of Common Factors (MCF) [35,36]	Sample question
Bond	Friendliness, acceptance, understanding, and support	Empathy, care, and genuineness	To what extent did you feel accepted by the program?
Partnership	Collaborative framework	Negotiation of goals, collaborative framework, and guidance	To what extent did you feel the program tried to influence you in ways that were helpful/not helpful?
Confidence	Respect for professional competence	Trust, development of a secure base, positive treatment expectancies	To what extent did you feel you could rely on the program for advice when you needed it?
Openness	Personal disclosure (client)	N/A	To what extent did you feel comfortable providing personal information to the program?
Client Initiative	Setting the agenda, client responsibility	N/A	To what extent did you feel the program allowed you to set your own goals?
Accessibility	N/A	Convenience and availability	To what extent did you feel the program was easy to use?
Reciprocity	N/A	Education and rationale giving, sensitivity, and flexibility	To what extent did you feel the program was flexible enough to meet your needs?

### Analysis Strategy

Quantitative analyses were conducted using SPSS version 21, and used data derived from participants who returned a post-intervention questionnaire. Internal consistency of the modified ARM subscales was tested using Cronbach’s alpha, and subscale means were examined to determine alliance strength. In line with previous studies of Internet-delivered therapies [22,23], residual gain scores were calculated to represent post-intervention treatment gains using the formula described by Steketee and Chambless [37] (the standardized subscale score at post-intervention minus the standardized subscale score at baseline, multiplied by the correlation between these scores). Residual gain scores thus represented treatment gains at post-intervention scores adjusted for baseline scores, and bivariate correlation analyses examined relations between these and scores on the ARM subscales. Since ARM data was collected at post-intervention only, it was not possible to perform the analyses on an intention-to-treat basis [24].

Interview transcripts were audio recorded, transcribed, and analyzed independently by 2 of the authors (MRB and MAB) using a word processing package (MS Word). Thematic analysis of responses was used to identify the main themes and subcategories, while at the same time a thorough search was conducted for divergent views to enable a richer description of therapeutic alliance features [38]. A 95% level of agreement was reached for themes and subcategories, with differences resolved after discussion. A total of 8 principal themes were identified and agreed upon, and these have been used as organizing themes for the qualitative data.

## Results

### Sample

**Figure 1:**
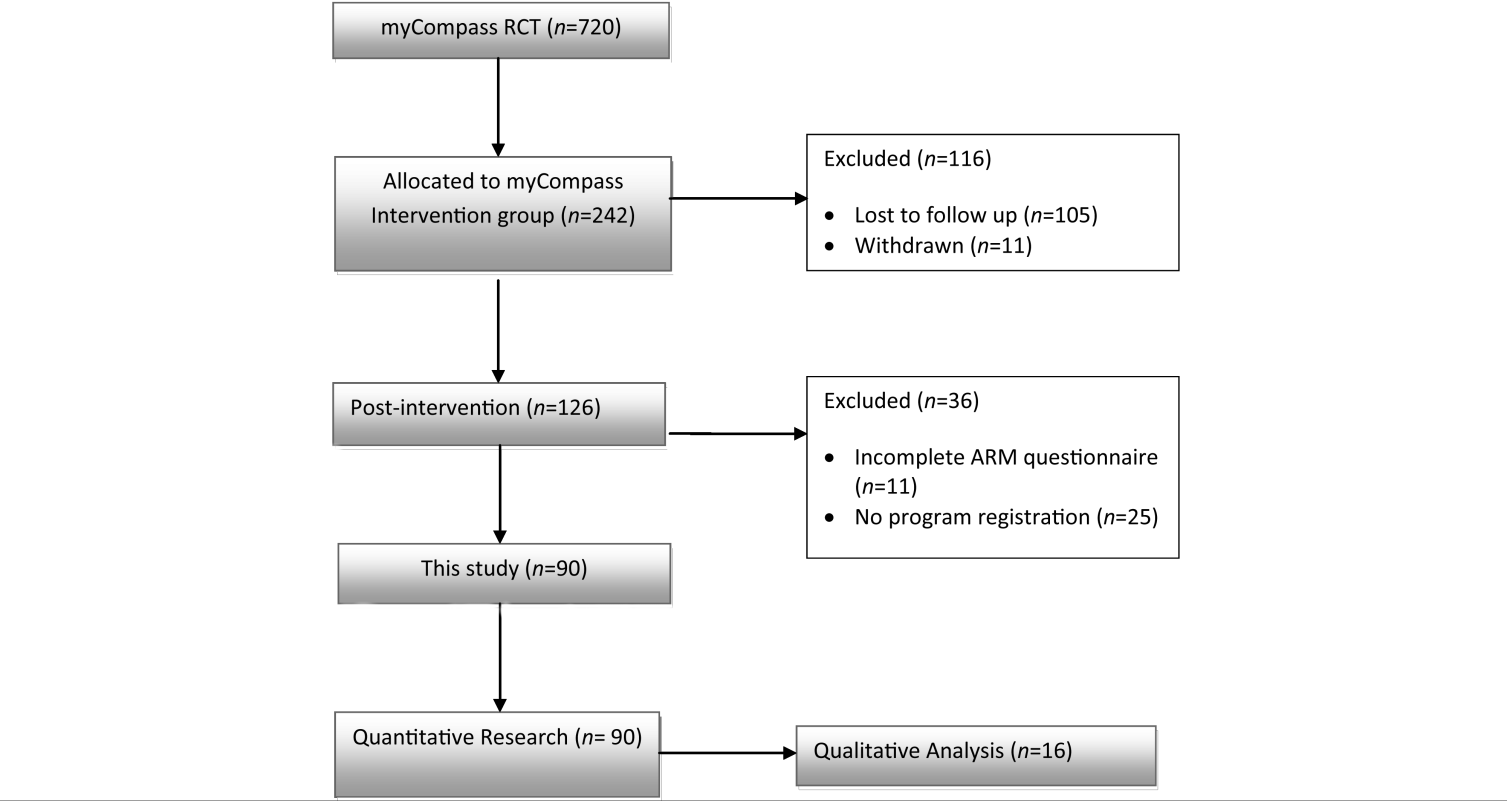
Study flow diagram.

Of the 231 RCT participants allocated to the myCompass intervention, 126 (54.5%, 126/231) returned post-intervention questionnaires (Figure 1). On closer inspection, we found that 11 (8.7%, 11/126) had not completed the ARM subscales, and a further 25 (10.8%, 25/231) had not registered with and did not use myCompass. We excluded data from both of these groups, leaving a final sample for this secondary analysis of 90 participants that were predominantly female (72%, 65/90), and employed (78%, 70/90), with a mean age of 38 years (SD 10 years) (Figure 1).

### Quantitative Results

#### Strength of the Therapeutic Alliance

Cronbach’s alpha coefficients for the ARM subscales ranged from .3 (client initiative) to .86 (confidence), and were slightly higher than those reported in Ormrod et al [26] (Table 2). The descriptive statistics for the ARM total and subscale scores are also shown in Table 2. All means were higher than the neutral midpoints (ie, 4), suggesting a positive therapeutic alliance with the myCompass intervention.

**Table 2:** Descriptive statistics for study outcomes at baseline and post-intervention (N=90).

Outcome		Baseline, mean (SD)	Post-intervention, mean (SD)	Chronbach alpha (scale^a^, scale^b^)
**DASS**				
	Depression	14.66 (9.68)	10.16 (7.92)	
	Anxiety	7.84 (6.68)	5.85 (6.80)	
	Stress	17.60 (7.63)	14.89 (7.51)	
	Total	40.10 (19.20)	30.91 (18.47)	
**WSAS**				
	Total	17.84 (8.93)	12.87 (7.99)	
**MHC-SF**				
	Emotional	2.84 (1.04)	3.35 (0.85)	
	Psychological	2.69 (1.02)	3.07 (1.04)	
	Social	2.09 (1.00)	2.51 (1.18)	
**ARM**				
	Bond	N/A	5.50 (1.03)	.82 (.82, .74)
	Client initiative	N/A	4.40 (0.92)	.30 (.55, .26)
	Partnership	N/A	4.71 (1.18)	.76 (.80, .59)
	Confidence	N/A	5.11 (1.15)	.86 (.87, .68)
	Openness	N/A	5.34 (1.17)	.74 (.77, .68)

^a^Scale reliabilities reported by Agnew-Davies and Stiles [27].

^b^Scale reliabilities reported by Ormrod et al [26].

#### Relations Between the Therapeutic Alliance and Study Outcomes

As in previous studies [27], we found that the bond, partnership, and confidence subscales of the ARM were highly inter-correlated (Table 3). For this reason, and to reduce the number of predictor variables, a composite score representing the average response across these subscales was computed for each participant to represent the overall quality of their emotional connection with the myCompass program [27].

**Table 3:** Correlations between the ARM subscales.

ARM subscale	Client initiative		Partnership		Confidence		Openness	
	*r*	*P* value	*r*	*P* value	*r*	*P* value	*r*	*P* value
Bond	.25	.01	.70	.00	.79	.00	.51	.00
Client initiative	N/A		.45	.00	.43	.00	.18	.08
Partnership	N/A		N/A		.72	.00	.27	.01
Confidence	N/A		N/A		N/A		.42	.00

Participant scores on the ARM subscales did not correlate with residual gain scores on the symptom, functioning, and positive well-being outcome measures (Table 4).

**Table 4:** Correlations between ARM subscales and residual outcome scores.

Outcome		Arm subscales					
		Composite score		Client initiative		Openness	
		*r*	*P* value	*r*	*P* value	*r*	*P* value
**DASS symptom scales**							
	Depression	-.07	.52	.14	.18	.08	.47
	Anxiety	.00	.99	.07	.52	.01	.95
	Stress	.020	.85	.20	.06	.08	.43
	Total	-.03	.81	.17	.11	.07	.48
**WSAS**							
	Total	-.19	.09	.04	.71	-.16	.13
**MHC-SF well-being scales**							
	Emotional	.18	.09	-.12	.25	.20	.06
	Psychological	.19	.07	-.02	.82	-.15	.16
	Social	.18	.08	-.19	.07	.01	.90

In contrast, participants’ ratings of the quality of their emotional connection with the myCompass program correlated significantly and positively with all 3 indices of program engagement. Furthermore, the client initiative (*r*=.26, *P*=.02) and openness (*r*=.25, *P*=.04) subscales were correlated significantly in a positive direction with self-monitoring frequency (Table 5).

**Table 5:** Correlations between ARM subscales and program engagement.

Engagement index (frequency)	ARM subscales					
	Composite score		Client initiative		Openness	
	*r*	*P* value	*r*	*P* value	*r*	*P* value
Program logins	.33	.00	.19	.06	.26	.11
Modules undertaken	.38	.00	.14	.19	.12	.57
Self-monitoring	.32	.00	.26	.02	.25	.04

### Qualitative Analysis

Interviews were conducted with 16 participants, 3 of whom were male with a mean age 40.1 years (SD 8.4 years). Thematic analysis elicited 8 themes that described participants’ relationship with the myCompass intervention (Textbox 1).

**Qualitative interview themes and subcategories.**

Themes and subcategories

- Empathy and acceptance

1. Felt supported and understood

2. Did not feel judged

3. Able to be oneself

- Working in partnership

1. Collaboration

2. Motivated goal attainment (eg, prompts and reminders)

- Confidence and reassurance

1. Respect for program content

2. Positive regard for skills and techniques

3. Expectations about program effectiveness

- Openness

1. Personal disclosure (client)

2. Privacy and/or confidentiality encouraged honesty

- Client initiative

1. Able to set one’s own agenda and/or goals

2. Flexibility to use the program in a structured and/or self-guided manner

- Availability

1. Convenience (eg, mobile phone, desktop)

2. 24/7 access

- Interactivity

1. Interactive exercises

2. Home tasks

3. Text message and/or email reminders and prompts

- Responsiveness

1. Program matched symptom needs

2. Personalized feedback

3. Graphical reporting of symptoms

#### Empathy and Acceptance

Interviewees commented that they felt accepted and supported by the myCompass program, and a majority felt that it offered a safe and non-judgmental context within which to deal with their difficulties.

The program was good, you could tell that it was made with an empathic voice, with no ulterior motives and that it was purely to help you. And you could tell that the authors had no judgment.

Female, 28 years

One participant reported that she did not feel the program understood or accepted her.

It’s just a computer...it doesn’t need to understand me...If it was a therapist it would be different.

Female, 27 years

#### Working in Partnership

All interviewees felt that they collaborated flexibly with the myCompass program to set and work towards achieving their treatment goals. For a majority of users, the automated alerts and reminders contributed to this partnership and were viewed as important motivators of goal achievement and prompts for staying on track.

When prompted, I responded to guidance to talk to a friend. I hadn’t realized how low I was at that time

Female, 48 years

#### Confidence and Reassurance

Overwhelmingly, interviewees expressed confidence in and respect for program content, and experienced this as reassuring. Furthermore, a majority were optimistic that the quality of the skills taught and advice provided improved their capacity to manage future mental health problems. Several interviewees reported that they were at first skeptical about the usefulness of the program, but noted rising confidence with increased program use.

The more modules I did, the more confident I felt in the information. It was becoming more and more helpful, especially the home tasks

Female, 41 years

One interviewee reported decreasing confidence in the program over time due to frustration caused by the compulsory home tasks.

#### Openness

Interviewees generally appreciated the privacy afforded to them by the program, and the opportunity to openly and honestly share their problem feelings and behaviors. Indeed, for some interviewees, the level of comfort they felt in interacting with the program was greater than they had experienced in the face-to-face context, including with friends and family.

It’s very easy when you’re feeling down to put on a brave face and tell everybody you’re fine...but with the computer I was comfortable being honest...Instead of trying to pretend that everything was fine, I could actually say it wasn’t...I felt more in control of things

Female, 47 years

#### Client Initiative

Some interviewees commented on the flexibility of the program in offering a structured (ie, making recommendations about symptom monitoring and modules), yet at the same time self-guided (ie, capacity for users to choose monitoring dimensions and modules) intervention. The capacity to choose how they used the program was generally viewed as empowering by the interviewees.

There’s a little quiz that you do when you start, and I was a bit surprised at some of the areas that it recommended for me to look at. Then I thought I’ll take a couple of those, and another one I was quite interested in as well, but ignore some of the ones that I thought weren’t so relevant...I felt quite able to make decisions about which ones I was going to look at.

Female, 42 years

The linear structure of the program’s modules was viewed negatively by a minority of interviewees who would have liked greater control over the speed with which they progressed through the interactive tasks.

#### Availability

A majority of interviewees accessed myCompass from both an Internet-enabled mobile phone and desktop computer; 3 interviewees chose not to access the program on their phone.

The availability of myCompass 24/7, and the option of using myCompass when and where needed from a mobile device were viewed as major program advantages by all interviewees. Nevertheless, some interviewees felt that myCompass was more easily used on desktop computers than mobile phones because of the larger screen size and more reliable Internet connectivity.

I liked the flexibility. And having it available on my mobile phone, just having the convenience there, meant that I could carry it around with me, I could update it on a needs basis, and it’s something I could do at a time and location of my choosing rather than being stuck at a desk

Male, 37 years

#### Interactivity

A majority of users commented on the usefulness of the interactive elements of the myCompass program, especially the in-task activities and the homework tasks. Several interviewees also appreciated being able to graph their self-monitoring data alongside contextual information.

myCompass helped me make changes...Noting my responses and reactions to certain situations and identifying those particular triggers that I wanted to monitor...There were things that I thought might be an issue for me, and it (the graphs) confirmed that they were

Female, 31 years

One interviewee commented that the feedback graphs provided tangible evidence that she was underestimating the extent of her symptoms.

It (the graphs) identified that perhaps when I was convincing myself that I was fine, that perhaps really I wasn’t.

Female, 47 years

Some users reported that the feedback provided to them when they logged into the program about their self-monitoring and module progress put extra pressure on them.

Sometimes I would log in and there were lots of red marks. I felt pressured...like I’d missed my school work

Female, 39 years

#### Responsiveness

Many users made comments about the capacity of the myCompass program to respond appropriately to their unique symptom needs, but views were mixed in this regard. On the one hand, there were users who felt the program recognized and appropriately responded to their personal symptom experience.

If I was having low days, it would...acknowledge that I’m having a low day and (that) it’s ok to have a low day and have you considered speaking to somebody. Instead of just going, you know what, I can’t help you

Female, 41 years

Conversely, some users commented that the program responded in ways that did not reflect an appreciation of their needs and left them feeling confused and misunderstood.

I tracked how I was feeling and then I’d get a message come up that says...perhaps you’re really struggling at the moment and you need to talk to somebody. And I’d think that I didn’t feel that I was that bad

Female, 46 years

## Discussion

### Principal Findings

This secondary analysis of data from a large RCT explored the extent and nature of the therapeutic alliance in the fully automated, mobile phone and Web-based intervention, myCompass. Consistent with Ormrod et al [26] and other studies of Internet-delivered therapies [21,22], results of the quantitative analysis showed that participants reported development of a positive alliance with the myCompass intervention. A point of differentiation from most previous studies, however, is that these findings were obtained in the absence of any therapist involvement in delivery of the intervention. Insights gained from the qualitative interviews provided further evidence that non-specific alliance features were experienced in participants’ interactions with the intervention, including empathy, collaboration, reassurance, and reciprocity. Whereas classic definitions of the therapeutic alliance require that a client and therapist are involved in the relationship [39], and therapists have previously questioned or down-played the existence of relationship process variables in computer-based therapies [19], our findings show that a significant and positive alliance can exist in the Internet environment in the absence of human support.

We also examined whether therapeutic alliance features were associated with treatment gains and program engagement. Consistent with previous studies of Internet-delivered interventions across a range of disorders [21,22,26], we found no support for a link between therapeutic alliance factors and symptom, functional, and positive well-being outcomes. These findings contrast with those for face-to-face psychotherapies [40], and lend further support for the idea that the quality of the alliance in the Internet environment may be less important than other factors for understanding treatment gains [21,26].

On the other hand, components of the therapeutic alliance did show significant and positive associations with participants’ level of engagement with the myCompass intervention. Most notably, ratings of perceived emotional connection with the program correlated positively with program logins, frequency of self-monitoring, and the number of psychoeducational modules completed, suggesting that users may engage with an Internet intervention to the extent that they experience a collaborative partnership that is working well, just as in face-to-face therapy [11]. Engagement with program content is generally linked with increased treatment gains, yet rates of adherence with Internet-delivered therapies are characteristically low [34], and attrition rates are high [41]. A contribution of our study, therefore, is to suggest that incorporating program content and functions that target alliance processes directly may improve client adherence and retention in the Internet context. Indeed, it has recently been proposed that the real value of a strong alliance may lie in its capacity to promote therapeutic engagement as opposed to contributing to clinical improvement [11].

Because the ARM and other popular measures of the therapeutic alliance (eg, the Working Alliance Inventory [42]) were originally developed for the face-to-face context, they are unlikely to tap clients’ perceptions of common factors or relationship features that may be distinct to Internet psychotherapies. For example, flexibility in the nature of the therapeutic encounter (in terms of time, location, and duration of access) is generally considered a particular advantage of Internet treatment [1], as is the option of interacting via different communication pathways [43]. However, these relationship variables are not assessed in existing alliance measures, and they remain largely unexplored as contributors to psychotherapy outcomes in the Internet context [1]. Further work is needed to conceptualize, from a client’s perspective, the common or non-specific processes that characterize relationships in the Internet environment, and to examine the implications of these for treatment outcomes and program engagement using appropriately developed and validated alliance measures.

### Limitations

Some limitations must be considered in interpreting these findings. As we have discussed previously [28], dropout attrition in the intervention arm of the RCT was high, especially among employed participants. Given positive links between alliance strength and treatment engagement [11], and a typical pattern of high alliance ratings among trial participants [23], we cannot discount the possibility that dropout from the intervention group reflected a lack of perceived alliance with the myCompass intervention. Furthermore, the sample was predominantly female which is potentially problematic as gender differences appear to influence the therapeutic alliance [44]. The generality of our findings, therefore, is somewhat limited.

From a measurement point of view, we remain uncertain as to whether the psychometric adequacy of the ARM is affected when scale items are adapted along the lines reported here (ie, replacing the word “therapist” with “program”). Until reliability and validity of the modified ARM is demonstrated and/or alternative measures of the human-computer alliance are developed, our conclusions must be considered tentative. In addition, therapeutic alliance data were collected at only one time point (ie, at post-intervention). Previous researchers have discussed the importance of measuring therapeutic alliance variables at various stages of the therapeutic process [25], and there is evidence that therapeutic success in the face-to-face context is more likely for patients whose alliance increases in the early stages of treatment [45,46]. At the same time, relations between therapeutic alliance features and program engagement are potentially reciprocal, such that a strong alliance predicts increased program interactions and vice versa [11]. Repeated administration of the ARM over the course of the intervention would have allowed a stronger test of the contribution made by therapeutic alliance features to treatment outcomes, program engagement, and study dropout.

### Conclusions

This study is among the first to provide quantitative and qualitative support for the existence of a positive therapeutic alliance with a fully automated, mobile phone and Web-based psychotherapy intervention involving no therapist assistance. Although it appears that a strong alliance contributes less proximally to therapy outcomes in a fully automated, Internet-delivered intervention compared with face-to-face psychotherapy, our data suggest that the ability to connect meaningfully and work collaboratively may be similarly important for client engagement across both contexts. Client engagement is vital for therapeutic success, yet effective translation of non-specific alliance components of face-to-face therapies into the Internet environment is not easily achieved [10]. For the sake of expediency, and in the interest of optimizing therapeutic gains, future studies should isolate the critical relational components of encounters in the Internet environment that relate to treatment outcomes and client engagement. These can then be honed in the development of new and refinement of existing Internet-delivered interventions.

## Appendix

Multimedia Appendix 1 CONSORT-EHEALTH checklist V1.6.2 [47].

## Abbreviations

ARM: Agnew Relationship Measure
CBT: cognitive behavior therapy
DASS: Depression, Anxiety and Stress Scale
MHC-SF: Mental Health Continuum-Short Form
RCT: randomized controlled trial
WSAS: Work and Social Adjustment Scale
